# Orthologous Transcription Factors in Bacteria Have Different Functions and Regulate Different Genes

**DOI:** 10.1371/journal.pcbi.0030175

**Published:** 2007-09-07

**Authors:** Morgan N Price, Paramvir S Dehal, Adam P Arkin

**Affiliations:** 1 Physical Biosciences Division, Lawrence Berkeley National Laboratory, Berkeley, California, United States of America; 2 Virtual Institute for Microbial Stress and Survival, University of California Berkeley, Berkeley, California, United States of America; 3 Department of Bioengineering, University of California Berkeley, Berkeley, California, United States of America; Duke University, United States of America

## Abstract

Transcription factors (TFs) form large paralogous gene families and have complex evolutionary histories. Here, we ask whether putative orthologs of TFs, from bidirectional best BLAST hits (BBHs), are evolutionary orthologs with conserved functions. We show that BBHs of TFs from distantly related bacteria are usually not evolutionary orthologs. Furthermore, the false orthologs usually respond to different signals and regulate distinct pathways, while the few BBHs that are evolutionary orthologs do have conserved functions. To test the conservation of regulatory interactions, we analyze expression patterns. We find that regulatory relationships between TFs and their regulated genes are usually not conserved for BBHs in Escherichia coli K12 and Bacillus subtilis. Even in the much more closely related bacteria Vibrio cholerae and Shewanella oneidensis MR-1, predicting regulation from E. coli BBHs has high error rates. Using gene–regulon correlations, we identify genes whose expression pattern differs between E. coli and S. oneidensis. Using literature searches and sequence analysis, we show that these changes in expression patterns reflect changes in gene regulation, even for evolutionary orthologs. We conclude that the evolution of bacterial regulation should be analyzed with phylogenetic trees, rather than BBHs, and that bacterial regulatory networks evolve more rapidly than previously thought.

## Author Summary

Living organisms use transcription factors (TFs) to control the production of proteins. For example, the bacterium E. coli contains a TF that prevents it from making enzymes that degrade lactose when lactose is absent. Bacterial genomes encode a huge diversity of TFs, and except in a few well-studied organisms, the function of these TFs is not known. To predict the function of a TF, biologists often search for a similar TF, from another organism, that has been characterized. It is generally believed that orthologous TFs—TFs that are derived from the organisms' common ancestor—will have conserved functions. The authors show that a commonly used method to identify orthologous TFs gives misleading results when applied to distantly related bacteria: the “orthologous” TFs are evolutionarily distant, they sense different signals, and they regulate different pathways. Biologists often predict, more specifically, that orthologous TFs will regulate orthologous genes. However, the authors show that even in more closely related bacteria, where the orthologous TFs do have conserved functions, these specific predictions are often incorrect. It seems that gene regulation in bacteria evolves rapidly, and it will be difficult to predict regulation in diverse bacteria from our knowledge of a few well-studied bacteria.

## Introduction

A fundamental goal of comparative genomics is to identify shared functional features between organisms and to elucidate the evolutionary divergence of those features. For example, when can information about gene regulation be transferred from model organisms to sequenced relatives? How does transcriptional regulation evolve in bacteria, and how do the transcription factors (TFs) themselves evolve [1–5]? Because a single bacterial genome may encode hundreds of TFs, including dozens of representatives of a single family of regulators, analyzing the evolution of these TFs is not straightforward. Indeed, we will show that a popular method in comparative genomics gives misleading results when applied to TFs.

The first step in most comparative genomics analyses is to identify corresponding genes, or orthologs. Orthologs are formally defined as homologous genes that diverged from a common ancestor by vertical descent [6]. Orthologs contrast to paralogs, i.e., genes that diverged by gene duplication, and xenologs, i.e., homologous genes whose history of divergence includes one or more horizontal gene transfer (HGT) events. As strictly defined, orthology describes the evolutionary relationships of genes, not their functional relationships. Thus, orthologous genes need not necessarily have conserved functions. Although orthology is often thought of as a one-to-one relationship between two genes from different organisms, evolutionary orthology allows for more complex relationships: for example, two recently duplicated genes in one organism are evolutionary co-orthologs of the corresponding gene from a lineage that did not experience the duplication event.

However, in studies of bacterial evolution, this evolutionary definition of orthology is not generally used. Because of high rates of HGT, many genes are xenologs rather than orthologs. Unfortunately, identifying all instances of HGT is quite difficult, and different methods give contradictory results [7]. Nevertheless, it is clear that HGT is widespread, and there is agreement that many or most of the genes in extant bacteria were acquired by HGT [8–10].

Instead of evolutionary orthology, most workers in the field use an informal concept of functional equivalence: orthologs are homologous genes that are closely related and are predicted to have the same or closely related function. Due to the lack of functional data for most genes, orthologs are defined as bidirectional best BLAST hits (BBHs) or are obtained from higher-level BLAST-based approaches such as collections of orthologous groups (COGs) [11]. This operational definition of orthology has the advantage that functionally corresponding genes can be considered as being equivalent regardless of the details of their evolutionary history. However, because most of the genes being considered are known only from the genome sequence, it is difficult to know if they actually have corresponding functions. Thus, using predicted functional equivalence as a basis for orthology has been criticized for lacking rigor [12]. Another problem with BBHs is that, because of uneven evolutionary rates, the best BLAST hit may not be the nearest homolog in the evolutionary tree [13].

We wondered how the concept and practice of orthology, whether defined by evolutionary analysis or by expected functional equivalence, applies to TFs. The most popular methods for assigning orthology are BBHs and COGs. The COG approach is less useful for studying TFs because it collapses large families of TFs into single orthology groups. For example, E. coli K12 has 45 members of the LysR group (COG583) that respond to a wide variety of signals and regulate different sets of genes.

On the other hand, many studies have used BLAST-based orthologs to study regulation in bacteria [2,4,5,14–16]. These studies assume that regulation is conserved, so that orthologous TFs will regulate orthologous genes. However, because bacterial genomes often contain many members of a TF family, we suspected that ortholog assignment by BBHs would often give misleading results. The presence of large families indicates that TFs often duplicate, and a best-hits approach will select the least-diverged duplicate as an ortholog. Also, if all of the close homologs have been deleted, then distant homologs will still be present, and one of these distant homologs may be a spurious BBH. In bacteria, paralogous TFs usually have different functions, and transcriptional regulatory networks can evolve rapidly [1,3,4], so in either case, the homolog's function will likely have changed. In contrast, evolutionary analyses of orthology consider all genomes, rather than just two at a time, so these errors can be avoided.

Nevertheless, BLAST-based orthologs have successfully been used to identify conserved regulatory sequences [2,14–16] and to link clusters of similar regulatory sequences (“motifs”) with the TFs that bind those sequences [17]. We argue that these analyses have succeeded because they focused on closely related bacteria, such as the family of Enterobacteria, within which duplication and change of function have had less time to occur. Indeed, conserved regulatory sequences are much more likely to be found when only close relatives are considered [18]. This illustrates another reason why BLAST-based orthologs have been effective in this context, which is that motif searching will usually return results only for those genes whose regulation is conserved. If the regulation has changed, then these methods will usually return no results, rather than incorrect results.

More recently, evolutionary studies have examined the conservation of TFs and their regulated genes across distantly related bacteria [4,5]. These studies used putative orthologs from BBHs to determine the presence and absence of TFs, and predicted that orthologous TFs will regulate orthologous genes if both orthologs are present. (These studies did not search for conserved regulatory sequences.) At the larger phylogenetic distances considered by these studies, BBHs might give misleading results.

Here, we test the BBHs of the characterized TFs of E. coli by both evolutionary and functional criteria. To test whether the BBHs are evolutionary orthologs, we built phylogenetic trees for all BBHs of TFs between E. coli and B. subtilis. To determine whether regulation is maintained between BBHs, we systematically examined characterized TFs and expression data from organisms at a range of phylogenetic distances from E. coli. We found that the orthology assignments for TFs from distantly related bacteria (e.g., bacteria from different divisions) are unreliable: more often than not, the BBHs are not evolutionary orthologs and have different functions. Furthermore, expression data suggest that regulatory interactions are often not conserved, even at closer phylogenetic distances and even when the genes concerned are evolutionary orthologs. We found experimental evidence in the literature and evidence in the genome sequence to confirm some of these changes. Thus, gene regulation appears to evolve more rapidly than previously thought.

## Results

### “Orthologous” Transcription Factors between E. coli and B. subtilis

**Figure 1:**
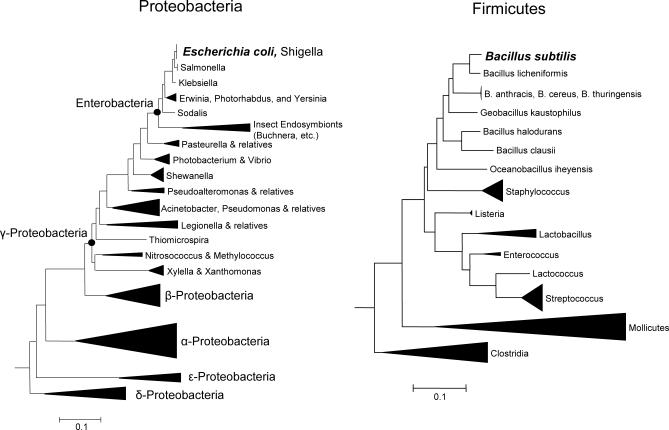
Phylogeny of Sequenced Members of the Proteobacteria and the Firmicutes

We first examined the putative orthologs, from BBHs, for TFs in E. coli K12 and B. subtilis. This comparison is convenient because dozens of TFs in each organism have been studied experimentally and because known regulatory interactions have been compiled [19–22]. Furthermore, many relatives of these bacteria have been sequenced (Figure 1). Although E. coli and B. subtilis are distantly related and belong to different phyla, recent evolutionary studies have considered “orthologous” TFs between them [4,5].

**Figure 2:**
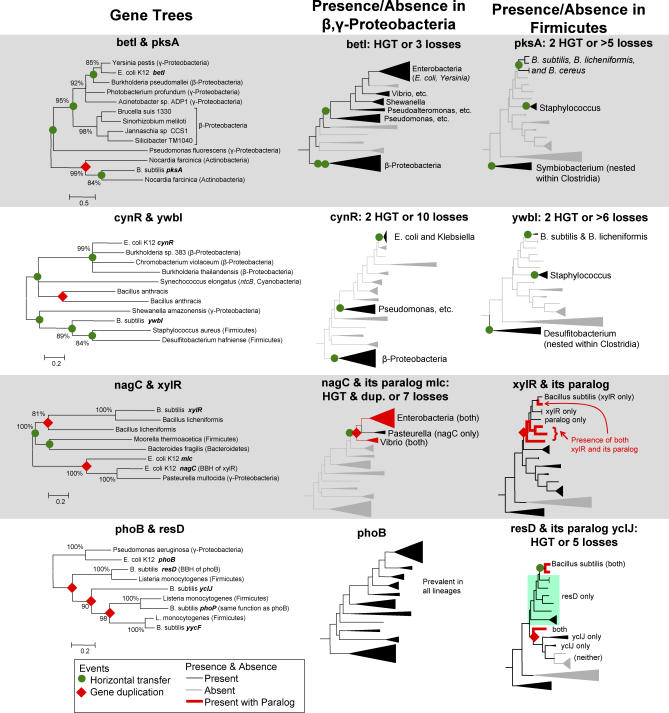
Evolutionary Histories of “Orthologous” Transcription Factors from E. coli K12 and B. subtilis (Left panels) For each pair of BBHs, we selected illustrative homologs and constructed a gene tree with TreePuzzle [43]. For confident clades, we show the support values. Close homologs from distantly related taxa are evidence for horizontal transfer events, and close homologs within one genome (i.e., paralogs) are evidence for duplication events. The rooting is arbitrary. (Center panels) The presence and absence of close homologs of the E. coli gene within β,γ-Proteobacteria, and the number of loss events required to explain the gene's distribution if HGT did not occur. (Right panels) Presence/absence of close homologs of the B. subtilis gene within Firmicutes.

Of the 159 E. coli TFs that are described in RegulonDB 5.6 [22], 35 have BBHs in B. subtilis. For these 35 BBHs and their homologs in other sequenced genomes, we examined their phylogeny and compared their functions (see Table 1 and Figure 2 for examples, and see Text S1 for a brief discussion of each BBH).

**Table 1:**
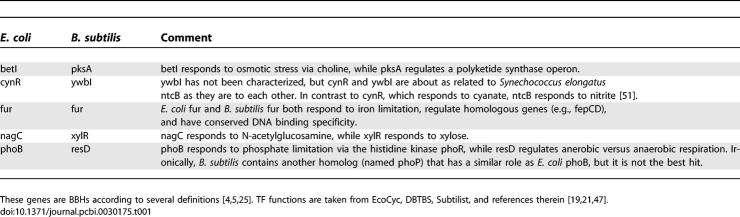
Functional Comparison of “Orthologous” Transcription Factors from E. coli K12 and B. subtilis

For 28 of the 35 BBHs, we found that they are not one-to-one evolutionary orthologs because there exist gene duplications and/or multiple HGT events that separate the two genes. We show four typical examples in Figure 2. First, betI and pksA are not evolutionary orthologs because both genes have complex evolutionary histories and at least three HGT events separate the two genes. The relationship between cynR and ywbI, or between nagC and xylR, also includes HGT events on one or both sides, as well as gene duplication events. Finally, E. coli phoB is the BBH of B. subtilis resD, but resD has paralogs that are probably more closely related to each other than they are to phoB. Because these genes belong to a large and diverse family, and because larger trees have poor support values (unpublished data), it is difficult to know if resD and its paralogs are evolutionary co-orthologs of phoB, although the HGT of yclJ is partial evidence against this. It is also possible that the root of the tree could lie between phoB and resD, so that they are evolutionary orthologs, but if distant family members (e.g., cpxR and ompR from E. coli) are included in the tree, then phoB, resD, and the paralogs all group together (unpublished data), which suggests otherwise. In any case, the presence of these closer paralogs shows that the BBH is misleading. Indeed, one of resD's paralogs (phoP) has a similar function as phoB, but because it is more diverged, it is not the BBH. Overall, 24 BBHs were rejected as evolutionary orthologs because the trees showed multiple events of HGT or gene duplication, including at least one HGT event, and four BBHs were rejected as one-to-one evolutionary orthologs purely because of closer paralogs.

Among the seven remaining BBHs, two of them (yjdG/yufM and yjeB/yhdE) show conservation within close relatives of B. subtilis and E. coli and seem to have been transferred between them, so we classified these as xenologs. Another BBH, birA, shows conservation within Bacilli and within γ-Proteobacteria (the division that contains E. coli), but it seems to have been transferred between Bacilli and Archaea [23]. Because of this ancient transfer event, we also classified birA as a xenolog. We classified the remaining four BBHs (dnaA, fur, lexA, rpoN/sigL) as evolutionary orthologs, although there may yet be ancient HGT events that we did not detect.

For 23 of the 35 BBHs, the B. subtilis member of the BBH (or a close homolog) has been studied experimentally. We defined a BBH as having a conserved function if both TFs respond to the same signal, or, if the signal is not known, we asked whether they regulate one or more corresponding pathways. We did not consider the TFs' DNA binding specificity or whether the regulated genes were homologous. Functional comparisons for five BBHs are given in Table 1. More broadly, as shown in Table 2, all of the one-to-one evolutionary orthologs have conserved functions. Xenologs that are separated by a single horizontal transfer event also had conserved functions. However, only two out of 14 characterized BBHs with more complex evolutionary histories have conserved functions (argR/ahrC and fliA/sigD). For cases where experimental evidence in B. subtilis is not available, evidence from other organisms often suggests that the functions are not conserved (e.g., cynR in Table 1). Although there is no reason why evolutionary orthologs must have the same function (in other words, a gene's function can diverge along the species tree), it is not surprising that genes with more complex evolutionary histories tend to have more diverged functions.

**Table 2:**
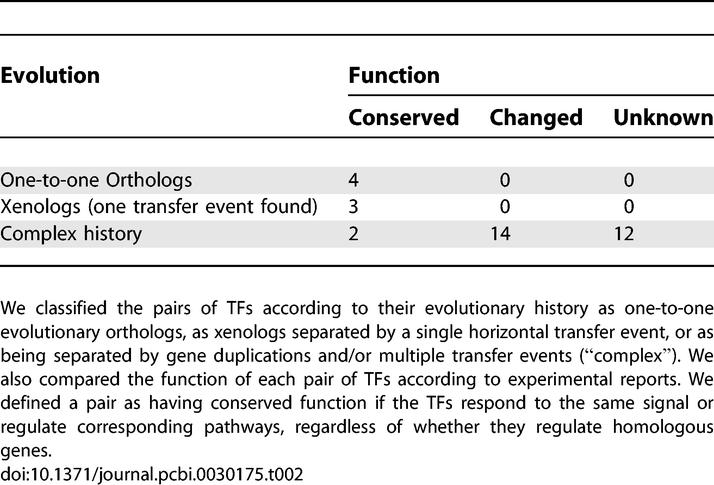
Evolutionary and Functional Categories of Bidirectional Best-Hit Transcription Factors from E. coli K12 and B. subtilis

Even when the TF's function is conserved, there are few cases where BBH TFs are known to regulate BBH genes. After excluding autoregulation, which is common for all TFs, we found only seven cases, and these involved only three of the 35 BBH TFs (fur, fliA/sigD, and argR/ahrC). We also found that cytR regulates deoC, and cytR's BBH ccpA regulates deoC's BBH dra, but because cytR and ccpA are not evolutionary orthologs and have unrelated functions, this seems to be convergently evolved rather than conserved from a common ancestor (see Text S1). It surprised us that there were so few conserved regulatory interactions, but it could be because of limited knowledge of transcriptional regulation in B. subtilis. The B. subtilis regulation database DBTBS includes only 35 regulatory interactions between a TF and a regulated gene whose BBHs in E. coli are described in RegulonDB. Overall, for genes that have BBHs and have been studied in both organisms, 20% of interactions (7/35) are known to be conserved.

### “Orthologous” Transcription Factors between E. coli and α,β-Proteobacteria

We next asked whether BBHs of TFs would have conserved functions at more moderate phylogenetic distances. We examined BBHs between E. coli and α,β-Proteobacteria. We used UniProt [24] and the MicrobesOnline database [25] to systematically identify characterized TFs from the α-Proteobacteria and β-Proteobacteria that are BBHs to characterized E. coli TFs. As shown in Table 3, of the 20 TFs that have characterized BBHs, nine regulate different pathways in these organisms than they do in *E. coli,* and another seems to have the same function but different DNA binding specificity.

**Table 3:**
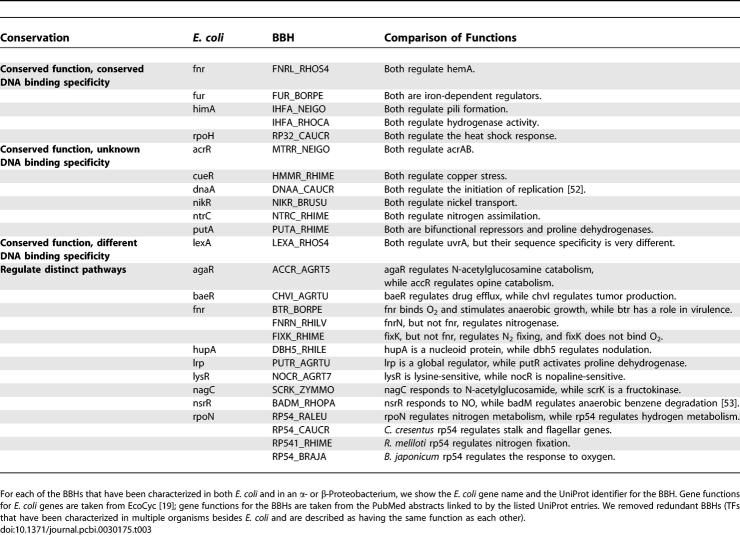
Characterized “Orthologs” of Transcription Factors between E. coli K12 and α,β-Proteobacteria

Although most of the functional differences seem clear-cut, the BBHs of rpoN illustrate the difficulty of defining “the function” of a global regulator. Because rpoN, or σ^54^, relies on co-activating TFs to sense signals, it can be involved in regulating a wide range of pathways. In E. coli, most of these pathways are nitrogen-related, so it is not surprising that some rpoN homologs (e.g., RP541_RHIME) regulate nitrogen fixation, which does not take place in E. coli. Other functions of the BBHs, such as regulating stalk and flagellar genes in Caulobacter crescentus, are more surprising.

We also examined some of the TFs that have been described in previous evolutionary studies as being conserved across distantly related bacteria [4,5]. (The details of this analysis are given in Text S2.) For example, it has been proposed that crp and fnr are global regulators throughout the prokaryotes, including Archaea, and that these have been conserved from the common ancestor [5]. However, both crp and fnr have characterized BBHs that have different functions (see Table 2 and Text S2).

**Figure 3:**
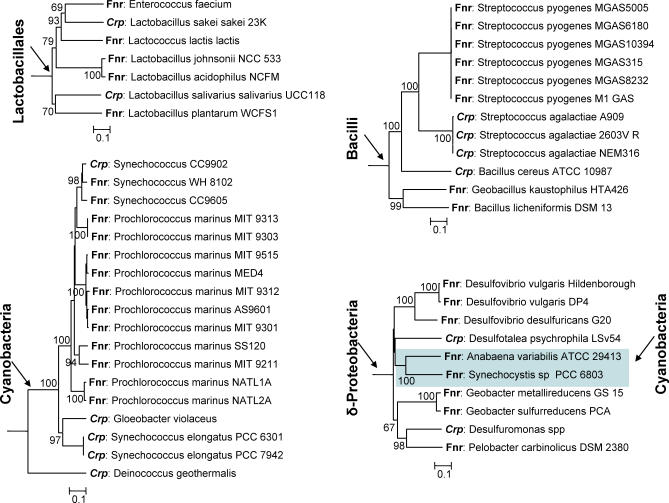
Bidirectional Best Hits of crp and fnr Are Not Orthologs We show selected clades from a phylogenetic tree of all BBHs of E. coli crp and E. coli fnr. For each gene, we show whether it is a BBH of crp or of fnr and what genome it is from. (The genome names include strain identifiers.) For each intermediate node, we show the bootstrap score (out of 100). The mixture of putative “crp” and “fnr” genes within well-supported clades shows that one or both assignments are incorrect. We also show which taxonomic group the genomes belong to. Except for the boxed Cyanobacteria, each clade in the gene tree corresponds to a taxonomic group in the species tree, which confirms that the gene tree is accurate and that the BBHs are misleading.

Another analysis reports that crp and fnr are present in an “alternating pattern” outside of the Proteobacteria [4]. We suggest that this is an artifact of BBHs: distantly related members of the crp/fnr family will have BLAST hits to both genes, and one of them will have a higher score and be a BBH, but which one has the higher score is not biologically meaningful. To confirm this hypothesis, we built a phylogenetic tree of all BBHs of crp and fnr from the MicrobesOnline database [25], using MUSCLE [26] and quicktree [27]. Within this tree, we looked for clades of genes that were from closely related species, which indicates that the genes are orthologs or recent paralogs, but which contained BBHs of both crp and fnr. We found clades with inconsistent BBHs within Lactobacillales, Streptococcus, Synechococcus, and δ-Proteobacteria (Figure 3). Because these clades contain genes from related organisms, errors in the phylogenetic tree are not a plausible explanation for these inconsistencies. Thus, BBH ortholog assignments for crp and fnr are misleading. This example also illustrates how errors from analyzing only two genomes at a time (as with BBHs) are easily apparent once more information is considered.

By searching for characterized homologs, we also found evidence that lrp, which is a global regulator of leucine levels in E. coli, arose within the Proteobacteria, rather than being an ancient global regulator [5]. Similarly, narL, which is proposed to be part of a conserved feed-forward circuit in both E. coli and in the α-Proteobacterium Rhodopseudomonas palustris [5], probably has a different function in R. palustris (Text S2).

Overall, we found that BBHs of TFs from different phyla or different divisions are usually not evolutionary orthologs. When characterized, BBHs from different divisions often have different functions (Table 3). However, the subset that are evolutionary orthologs often do have conserved functions (Table 2). We conclude that phylogenetic analysis is required to understand the evolution of TFs across distantly related bacteria.

### Orthology-Based Predictions of Transcriptional Regulation Are Not Reliable

After identifying orthologous TFs by BBHs, previous studies have predicted that these orthologous TFs would regulate orthologous genes (if such genes are present) [4,5]. This assumption has been used to transfer known regulatory interactions in E. coli K12 to other genomes, including distant relatives such as B. subtilis. Although such conservation of function seems unlikely to hold for the TFs we discussed above, we tested the quality of these predictions more broadly by using expression data.

**Figure 4:**
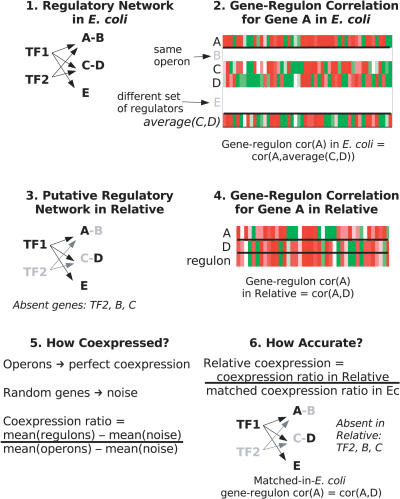
Using Patterns of Gene Expression To Test Putative Regulation in Relatives of E. coli (Steps 1–4) We use “gene–regulon” correlations to see if the regulation agrees with microarray data, both in E. coli and in its relatives. To compute gene–regulon correlations, we first average the expression pattern (the log-ratios) for other genes that are not in the same operon and are regulated by the same set of TFs. The rationale for using gene–regulon correlations instead of gene–gene correlations is explained in Methods. If the gene–regulon correlation is high in E. coli and low in the relative, then the regulation may have changed. (Step 5) To correct for the varying quality and quantity of the microarray data for the different species, we use operons as a positive control and random pairs of genes as a negative control. Thus, the “coexpression ratio” should depend on the accuracy of the predictions, and not on the quality of the microarray data. (Step 6) To quantify how the predictions compare with regulation in E. coli, we use the “relative coexpression.” To control for the absence of genes in the related organism, we compare the coexpression ratio in the related organism to a “matched” coexpression ratio that uses only E. coli genes that have BBHs in that organism.

**Figure 5:**
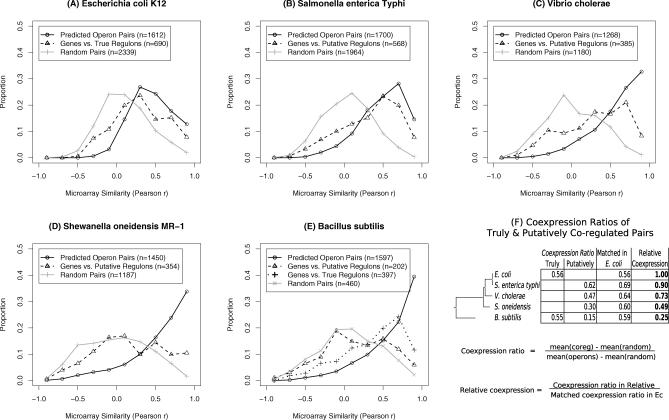
Coexpression Analysis Shows That Predicting Transcriptional Regulation from BBHs of Known Regulation Is Not Reliable (A) The distribution of gene–regulon correlations in E. coli. As a positive control, we show the correlation between adjacent genes that are predicted to be in the same operon. As a negative control, we show the correlation between random pairs of genes. (B–E) Correlations between genes and their putative regulons in other species, as predicted from BBHs of E. coli genes and TFs. The controls are as in (A). For B. subtilis (E), we also show gene–regulon correlations from known regulation in that organism [21]. (F) Phylogeny of the organisms and summary statistics that compare the predictions to true coregulation in E. coli and B. subtilis. See Figure 4 for more detailed definitions.

Specifically, we considered “regulons,” which we defined as E. coli genes that are reported in RegulonDB 5.6 to be regulated by the same set of TFs [22]. For each gene in each regulon, we computed a “gene–regulon correlation,” which we defined as the Pearson correlation coefficient between the expression pattern of the gene and the average expression pattern of other genes in the regulon (see Figure 4 and Methods). We only compared genes with other genes that are regulated by the same set of TFs because complex regulons give stronger coexpression than simple regulons [28,29]. Furthermore, because genes that are in the same operon will have similar expression patterns even if the predicted regulation for the genes is incorrect, when computing the average expression for the regulon, we considered only genes that are not likely to be in the same operon as the target gene. More precisely, we excluded genes if they are predicted to be in the same operon as the target gene [30] or if they are on the same strand as and are within 10 kilobases of the target gene. We computed these gene–regulon correlations for known regulation in E. coli and B. subtilis [21,22] and for putative regulons in other species that were predicted from the BBHs of E. coli genes and TFs. As expected, gene–regulon correlations in E. coli are on average higher than the correlations between random pairs of genes, but not as high as the correlations between genes that are in the same operon (Figure 5A).

Similarly, gene–regulon correlations for known regulons in B. subtilis are well above random (Figure 5E). In contrast, putative regulons in B. subtilis that were predicted from BBHs of E. coli genes have low gene–regulon correlations, and the distribution resembles that of random pairs of genes rather than that of truly coregulated genes (Figure 5E). As shown in Figure 5F, we summarized the distribution with the “coexpression ratio,” which ranges from 0 (the coexpression of random pairs of genes) to 1 (the coexpression of operon pairs). The truly coregulated genes in B. subtilis have a coexpression ratio of 0.55, while the putatively coregulated genes have a coexpression ratio of only 0.15. Thus, the expression data for B. subtilis is consistent with our previous conclusion that most of the regulatory interactions inferred for B. subtilis by BBHs are incorrect.

We also examined predictions for the γ-Proteobacteria *S. enterica typhi*, V. cholerae, and S. oneidensis MR-1. These species are much more closely related to E. coli than B. subtilis is, and most of the BBH TFs between these species and E. coli are evolutionary orthologs. For example, of 57 BBH TFs between E. coli and S. oneidensis MR-1, 40 are evolutionary orthologs according to an automated method [31]. In V. cholerae and S. oneidensis MR-1, gene–regulon correlations were much higher than for the B. subtilis predictions but much lower than for true E. coli regulons (Figure 5C and 5D). S. enterica gene–regulon correlations were about as high as in E. coli (Figure 5B). Thus, the strength of the gene–regulon correlations decreases as the phylogenetic distance from E. coli increases (Figure 5F). This trend remains after we compare the strength of coexpression for the predicted regulatory network in, e.g., S. oneidensis, with that of the subset of the E. coli network that has BBHs in S. oneidensis (see “matched in E. coli” and “relative co-expression” in Figure 5F). For all of these species, the expression data shows a strong difference between operon pairs and random pairs (all *p* < 10^−15^, Kolmogorov-Smirnov test; see Figure S1 for the cumulative distributions). Thus, the expression data is capable of detecting coexpression.

Our interpretation is that the predicted regulatory interactions in B. subtilis are mostly erroneous, and that even in S. oneidensis, which is much more closely related to E. coli and might be expected to conserve regulation, the predictions are not reliable. One caveat in our analysis is that a gene–regulon correlation might be low not because of an erroneous prediction for the gene of interest but instead because of erroneous predictions for other members of the regulon, and so the coexpression ratio might understate the accuracy of the predictions. Another caveat is that for genes that are under complex control, the loss of regulation by a single TF might eliminate most of the coexpression, even if the other regulatory predictions for that gene are correct.

To convert the relative coexpression ratio of 0.49 in S. oneidensis (see Figure 5F) to an error rate, we took the subset of the E. coli network that has BBHs in S. oneidensis and randomly shuffled a varying proportion of it to introduce “errors.” We then computed the relative coexpression for these “predictions” in E. coli as compared with the unshuffled (true) regulation. We found that the relative coexpression ratio of 0.49 corresponds to about 65%–80% of individual regulatory interactions being correct, or about 45%–75% of genes having all of their predicted regulation being correct (Figure S2). So, it appears that the relative coexpression corresponds roughly to accuracy at the gene level, and that the predictions for S. oneidensis do have a high error rate. These error rates are consistent with a phylogenetic footprinting study that included E. coli K12 and S. oneidensis MR-1 and reported that only 35%–70% of BBH promoters contained conserved sequences [18].

Another caveat in our analysis is that coexpression may be conserved even when the mechanism of regulation has changed. For example, S. oneidensis does not contain close homologs of either the E. coli purine regulator purR or its paralog rbsR. Nevertheless, 20 of the E. coli genes that are regulated by purR have evolutionary orthologs in S. oneidensis [31] and these genes are strongly coexpressed in S. oneidensis, with an average gene–regulon correlation of 0.59. Although in this case no predictions were made, this example nonetheless illustrates how relative coexpression can underestimate the error rate.

Our finding that predicted regulons in V. cholerae are only 73% as coexpressed as the true regulons of E. coli contrasts to a previous report by Babu et al. [5]. They conducted a similar test of BBH-based predictions for V. cholerae and claimed that the predicted co-regulated pairs are about as co-expressed as in E. coli. They used gene–gene correlations rather than gene–regulon correlations, and they used a different set of regulatory interactions, but we also found weaker coexpression when we used gene–gene correlations and when we used their set of regulatory interactions (unpublished data). It appears that they included pairs of genes that are in the same operon in their set of within-regulon pairs. Including such pairs would have greatly increased the number of strongly correlated predictions in V. cholerae (unpublished data). Genes that are in the same operon will be coexpressed regardless of whether the predicted regulation for the operon is correct, and so should not be used to test the accuracy of the predictions.

### Differences in Gene Regulation between E. coli K12 and S. oneidensis MR-1

To see whether low gene–regulon correlations in S. oneidensis reflect changes in gene regulation, we examined individual genes that were strongly correlated with their regulon in E. coli but were not at all correlated with their putative regulon in S. oneidensis. We used arbitrary thresholds of *r* > 0.5 in E. coli and *r* < 0 in S. oneidensis. Given these thresholds, we found 28 genes in 12 operons with a change in expression, which comprised 8% of the genes for which we predicted regulation in S. oneidensis. All but five of the 28 genes are in an operon (in E. coli) with another gene that has changed regulation, which shows that these changes are not due to noise in the microarray data. When we examined these operons, we found a variety of evidence that the difference in gene expression patterns reflects a true difference in gene regulation in S. oneidensis, which we summarize below. For a detailed discussion of each operon, see Text S3.

First, seven of the 12 operons are regulated in E. coli by arcA and/or by fnr. These regulators are evolutionary orthologs between E. coli and S. oneidensis (unpublished data), but they are reported to have different roles in the two organisms [32,33]. For example, the sdh operon is regulated by arcA in E. coli but not in S. oneidensis [32], as predicted by our gene-expression analysis. Furthermore, in E. coli, arcA's activity is controlled by the sensor histidine kinase arcB, but arcB is not present in the S. oneidensis genome. Because S. oneidensis can use a much wider variety of electron acceptors than E. coli can, it is not surprising that these global regulators of anaerobic or microaerophilic growth would have evolved different roles. These examples also illustrate how evolutionary orthologs can have somewhat different functions.

Second, a few of the regulated genes, and one of the regulators (fliA), are not evolutionary orthologs—the S. oneidensis genes are distantly related to their E. coli counterparts, and the BBHs are separated by multiple HGT events. Given this complex evolution, it is not surprising that regulation would not be conserved. In the case of fliA, experimental reports from other γ-Proteobacteria [34,35] confirm that the regulon is not conserved across these HGT events (see Text S3 for details).

Third, in several cases, we found evidence for a change in operon structure, so that the S. oneidensis gene is not expressed from the same promoter as in E. coli. We have previously shown that changes in operon structure often lead to changes in gene expression patterns [36]. For example, the E. coli operon argCBH is regulated by argR. In S. oneidensis MR-1, the gene cluster has expanded to argCBFGH. There is a strong and conserved putative argR binding site upstream of S. oneidensis argB (see Text S3), and argC is not coexpressed with argB in S. oneidensis MR-1 (*r* = −0.21). Both of these suggest that argB has its own promoter in S. oneidensis.

Fourth, as illustrated by argC, more careful examination of the microarray data itself can allow greater confidence that the change in expression patterns is biologically significant. As another example, in E. coli the clpPX operon is transcribed from an rpoH-dependent promoter, and clpP and clpX are considered to be heat shock genes. In S. oneidensis, however, both clpP and clpX are slightly downregulated in response to heat shock [37]. Furthermore, we did not find a plausible binding site for rpoH upstream of S. oneidensis MR-1 clpP (unpublished data).

Overall, of the 12 operons that we identified by gene expression analysis as having different regulation S. oneidensis than in E. coli, we found confirmatory evidence for nine operons.

## Discussion

We have systematically examined “orthologs,” as identified by BBHs, of characterized TFs from E. coli K12. We examined all BBHs of TFs between E. coli and B. subtilis, all BBHs of characterized TFs between E. coli and α,β-Proteobacteria, and in the gene expression analysis, all BBHs of TFs between E. coli and closer relatives at varying phylogenetic distances within the γ-Proteobacteria. We found that in bacteria from different divisions or different phyla, these BBHs are often not true evolutionary orthologs. Although the few BBHs that are evolutionary orthologs usually have conserved functions, BBHs with complex evolutionary histories usually have different functions: they respond to different signals and regulate different pathways. Even when the BBHs are one-to-one evolutionary orthologs and respond to the same signals, there are only a few cases where the BBHs regulate orthologous genes.

When we examined orthologs of TFs between E. coli and other γ-Proteobacteria, we found that, at this closer evolutionary distance, most of the BBHs are evolutionary orthologs, but they regulate different genes. In particular, we found that using orthology to predict transcriptional regulation has significant error rates. We estimated an error rate of about 30% for predicting regulation in S. oneidensis MR-1 based on BBHs to E. coli, and because coexpression can be conserved even when the mechanism of regulation has changed, the actual error rate may be higher. Although orthology is not, by itself, a reliable predictor of transcriptional regulation, it could probably be combined with comparative analysis of promoter regions and with expression analysis [14,16,23,38–40] to give more accurate results.

In any case, our results suggest that gene regulation evolves rapidly. We also identified nine high-confidence cases where the regulation in E. coli is not conserved in S. oneidensis. Many of these cases involve evolutionary orthologs, and, in all of these cases, the BBHs of the TF and the regulated gene probably have similar functions. Given more expression datasets and TF location data, it should soon be possible to identify large numbers of regulatory changes. It will be interesting to see what the functional consequences of these regulatory differences are and whether the differences in gene regulation are adaptive.

Our findings support the conclusion that “bacterial regulatory networks are extremely flexible in evolution” [4]. Because the use of BBHs has concealed many cases of flexibility by creating false orthologs out of regulators that play different roles, the true rate of evolution must be even higher. More broadly, it is proposed that regulatory networks evolve by “embedding orthologous genes in different types of regulatory motifs” [5]. However, given the high rates of false orthologs from BBHs, we suspect that the horizontal transfer, duplication, and loss of TFs are dominant features in the evolution of bacterial TFs.

It is less clear how the methodological problems we have discussed here affect other conclusions from previous evolutionary studies that relied on BBHs. It is reported that there is little [5] or no [4] tendency for TFs to be maintained or lost together with their regulated genes. However, while performing detailed phylogenetic analyses of E. coli regulators, we found more than 20 cases where a TF has been horizontally transferred together with adjacent genes that it regulates (Price, Dehal, and Arkin, unpublished). We suggest that false positives from BBHs are sufficiently common to conceal this effect.

Similarly, these authors find little difference between global regulators and more specific regulators in their tendency to be conserved. In contrast, we argue that global regulators evolve rapidly, but other regulators evolve even more rapidly. Our phylogenetic analyses suggest that most of the global regulators in E. coli have been inherited vertically since the divergence of E. coli and S. oneidensis, but most other TFs in E. coli have arisen by HGT or gene duplication after that divergence (Price, Dehal, and Arkin, unpublished). Again, false orthologs could be concealing the true difference.

Our results also imply that it will not be possible to predict the precise function of TFs in bacterial genomes by homology alone. Because of the rapid divergence of TF function, most annotations from BBHs are probably incorrect. This kind of error affects model organisms as well: for example, B. subtilis msmR appears to be named from a rather distant homolog in Streptococcus mutans (Text S1). Evolutionary analyses can identify most of the functional orthologs among the BBHs, but these are few. For example, of the 159 characterized TFs in RegulonDB 5.6, 35 have BBHs in B. subtilis, just nine of those have conserved functions, and seven of those nine are evolutionary orthologs or simple xenologs. For α,β Proteobacteria, which are more closely related to E. coli, there are about 40 BBH TFs per genome, and about half of the BBHs are functional orthologs, which would give about 20 predictions per genome. A computational study of the TFs in Lactococcus lactis, which is related to B. subtilis, also found that the specific functions of most TFs could not be predicted [1]. Thus, additional methods (e.g., [17,41]) will be needed to predict the functions of TFs across the diversity of bacteria.

## Methods

### Bidirectional best hits.

BBHs were taken from the MicrobesOnline database [25]. MicrobesOnline requires that the BLAST hit cover 80% of both genes, with e-values of at most 10^−5^ at an effective database size of 10^8^. In contrast, other workers require the domain structure of the protein to be unchanged [4]. The two definitions give very similar results for TFs, both because the coverage requirement usually forces the domain structures to match, and because TF families are so large that best hits usually have the same domain structure. Only one of the 35 BBHs between E. coli and B. subtilis that are tabulated in Table 2 have different domain structures (atoC/rocR).

### Evolutionary analyses.

To examine the evolutionary history of these BBHs, we used phylogenetic trees for COGs and other gene families from the MicrobesOnline tree-browser (http://www.microbesonline.org/treebrowseHelp.html). These trees, many of which contain thousands of sequences, were computed with a fast implementation of neighbor-joining [27]. We then built more accurate gene trees for selected homologs using CLUSTALW [42] and TreePuzzle [43], and compared the gene tree with the MicrobesOnline species tree. The MicrobesOnline species tree is a supertree of maximum likelihood trees of concatenated proteins (http://www.microbesonline.org/treebrowseHelp.html#speciestree).

We inferred a gene duplication whenever a clade contained more than one gene from an organism. We inferred a HGT event whenever genes from distantly related bacteria formed well-supported clades (e.g., betI in E. coli K12 and *Burkholderia*, in Figure 2). We also inferred HGT from the presence of close homologs in related bacteria and the absence of close homologs in species of intermediate relatedness (e.g., the absence of betI from distant γ Proteobacteria in Figure 2). In these cases we required that at least two loss events would otherwise be required to explain the pattern of presence/absence across species [44–46]. We also required that a small change to the gene tree (e.g., a single interchange) would not eliminate the putative HGT event. Because most HGT events are across greater distances, using a higher threshold for the number of loss events would rarely affect a gene's classification (e.g., examples in Figure 2). Also, we did not count losses in the highly reduced genomes of the insect endosymbionts (Buchnera, Candidatus Blochmannia, and Wigglesworthia) against this threshold of two losses.

Another issue in interpreting the gene trees is that the root of these trees is unknown. In the presence of HGT, there is no reliable way to choose an outgroup, and so the root of the tree cannot be determined. However, if our goal is to determine if HGT has occurred, then we can assume that HGT has *not* occurred and try to falsify that hypothesis. Thus, we used the E. coli gene as an outgroup for identifying HGT into the Firmicutes, and we used the B. subtilis gene as an outgroup for identifying HGT into the Proteobacteria. Even if the implied rooting is incorrect, this will only affect the direction of the HGT event, and not the existence of HGT.

To identify gene duplication events, we assumed that distant relatives of the genes (i.e., relatives that are more diverged than the BBHs are from each other) could be used as an outgroup. Because most of the TFs that we considered belong to large families, it is unlikely that the root that was implied by this method was misleading. In particular, it is not likely that the root would lie between the paralogs, in which case the BBHs could be one-to-one evolutionary orthologs. (For example, resD and phoB in Figure 2 could be evolutionary orthologs if the true root lies between resD and yclJ.) In general, we cannot rule out this possibility. If the common ancestor of the paralogs was acquired by HGT, however, as in the case of nagC and mlc (Figure 2), then a root between the paralogs becomes implausible, as it implies that the gene family was invented recently within a single group of bacteria, was highly conserved and duplicated within those bacteria, and yet proliferated by HGT and rapid divergence to many other groups of bacteria.

### Identifying characterized TFs.

To identify characterized TFs in E. coli, we used RegulonDB [22] and EcoCyc [19]. To identify characterized TFs in B. subtilis, we used DBTBS, Subtilist, and literature searches [21,47]. To find characterized TFs in S. oneidensis MR-1, we used RegTransBase [48]. For other organisms, we used the citations in UniProt entries, but we ignored papers that were cited for more than five genes, such as genome sequence papers.

### Why use gene–regulon correlations?

To estimate the accuracy of gene regulation predictions from microarray data, we used gene–regulon correlations. Gene–regulon correlations remove noise by averaging over the expression patterns of genes in the putative regulon, they allow us to determine which genes have changed regulation, and they eliminate a bias toward large and weak regulons that would arise from the naive use of gene–gene correlations.

First, gene–regulon correlations remove noise. For example, suppose that a predicted regulon is noisy, and contains four true positives and four false positives. When we average these expression profiles together, the contribution of the false positives will cancel out, because they are (probably) not correlated with each other. Thus, the four true positives with consistent expression will dominate the average, and so the average will be roughly correct, albeit with reduced intensity because half of it is from the false positives averaging each other out. When we compute the gene–regulon correlation, however, the correlation coefficient ignores the intensity of the input vectors, and so the gene–regulon correlations will (mostly) reflect the similarity of each gene to the true positives. We could average over gene–gene correlations instead, but that merely averages the noise, it doesn't reduce it. In our hypothetical scenario, 3/4 of the gene–gene comparisons will give low correlations, while only 1/2 of the gene–regulon correlations will give low correlations.

Second, gene–regulon correlations are interpretable. In the above example, half of the genes will still have high gene–regulon correlations, while from gene–gene correlations, most of the gene–gene correlations would be low, and it would not be clear which genes' expression patterns have changed. In practice, most of the gene–regulon correlations are computed using genes from several other operons (unpublished data), so these benefits of gene–regulon correlations are relevant.

Third, gene–gene correlations are biased toward large regulons, because the number of pairs within a regulon grows as the square of the regulon's size. For example, if we examine the coexpression of pairs of E. coli genes that are regulated by the same set of TFs and are not likely to be in the same operon, then for 29% of the pairs, both genes are regulated only by rpoE and not by any other TFs, at least as far as is reported in RegulonDB. In contrast, genes that are regulated only by rpoE account for only 9% of the gene–regulon correlations. The pairs of genes that are regulated only by rpoE are not correlated with each other—the mean gene–gene correlation is only 0.06, while the mean of the other gene–gene correlations within regulons is 0.20. Thus, if we used distributions of gene–gene correlations to examine the quality of predictions, almost a third of the distribution would be noise from this source, while with gene–regulon correlations, only a tenth of the distribution is noise from this source. This illustrates how using gene–regulon correlations reduces the effect of large regulons that are not coexpressed.

### Microarray data.

Microarray data for E. coli K12, S. oneidensis MR-1, and B. subtilis were taken from a previous compilation [36]. Microarray data for V. cholerae was taken from SMD [49]. Microarray data for *S. enterica Typhi* was taken from GEO [50]. This gave 212 experiments for E. coli, 37 experiments for *S. enterica Typhi*, 102 experiments for V. cholerae, 24 experiments for S. oneidensis MR-1, and 45 experiments for B. subtilis. Despite this variation in dataset size, the extent of the difference between operon pairs and random pairs was about the same in all five datasets (Kolmogorov-Smirnov D-statistic = 0.53–0.61).

For all of the microarray datasets, we analyzed normalized log-ratios (that is, the logarithm of the estimated fold-change in RNA levels between two conditions). Normalization methods are described in the data sources. We also subtracted the mean log-ratio from each experiment before we calculated correlation coefficients between genes. We only computed correlation coefficients for pairs where both genes had data for at least ten experiments. To compute gene–regulon correlations, we also required at least ten experiments, and we only used experiments that had data for both the gene and for at least half of the other members of the regulon.

## Supporting Information

Figure S1 Cumulative Distributions for Gene Expression Correlations for Adjacent Genes in Operons, for Gene-Regulation Correlations, and for the Correlation of Random Pairs of Genes This is the same data as in Figure 5, but showing the cumulative distribution rather than the histogram.
(46 KB EPS)

Figure S2 The Relative Coexpression Ratio for Regulatory Networks with Errors We introduced random changes into the E. coli regulatory network and measured the relative coexpression ratio. We used only the subset of the E. coli network that has orthologs in S. oneidensis MR-1 so that we could compare these to the coexpression ratio in S. oneidensis (which was 0.49). On the left, we show the coexpression ratio as a function of the fraction of regulatory interactions that were maintained. On the right, we show the coexpression ratio as a function of the fraction of genes whose regulation was unchanged. The straight line shows *x* = *y*, and each error bar shows the standard deviation over 20 randomized networks. The horizontal line shows the relative coexpression ratio for S. oneidensis.
(12 KB EPS)

Text S1 Comments on the 35 BBH TFs from E. coli and B. subtilis
(66 KB PDF)

Text S2 Differences in Function for Other BBHs (32 KB PDF)

Text S3 Regulatory Differences between E. coli and S. oneidensis MR-1 (53 KB PDF)

## Abbreviations

BBH: bidirectional best BLAST hits
COGs: collections of orthologous groups
HGT: horizontal gene transfer
TF: transcription factor
