# Supplementary material for: Orthologous Transcription Factors in Bacteria Have Different Functions and Regulate Different Genes
Source: PLoS Comput Biol. 2007 Sep 7;3(9):e175. doi: 10.1371/journal.pcbi.0030175 (PMC1971122; doi:10.1371/journal.pcbi.0030175)
Supplement: Supplementary file 1 [file pcbi.0030175.sd001.pdf]

Supplementary Text S1: Comments on the 35 BBH TFs from E. coli and B. subtilis

TF functions are taken from EcoCyc, DBTBS, Subtilist, and references therein (1; 2; 3). For *B. subtilis* genes, we also searched the literature (citations given below). The *E. coli* gene name is listed first. To view the evolutionary history of these genes, visit http://microbesonline.org, search for the *E. coli* or *B. subtilis* gene by name, and click on "T" for tree-browser.

Conserved function, evolutionary non-orthologs

fliA/sigD – These are also known as σ^{28}. Both genes regulate motA and flgB. sigD also regulates autolysis. Both genes show a complex history of HGT. fliA has being transferred between Enterobacteria and Azotobacter vinelandii (a distant γ-Proteobacterium) and various β-Proteobacteria. Close relatives of sigD are present in Bacilli (e.g. *B. clausii* and *Oceanobacillus iheyensis*) but not in most other Firmicutes. sigD also seems to show HGT within Bacilli (the gene tree is significantly different from the species tree).

argR/ahrC – Both are regulators of arginine metabolism, via binding to L-arginine, and both regulate argC. Sequence analysis has shown conservation of argR and of its DNA binding specificity across diverse bacteria (4). argR is absent from almost all of the Proteobacteria except for the γ-Proteobacteria, which suggests that it was acquired by HGT. *B. subtilis* ahrC is adjacent to recN, and this arrangement is conserved in most Firmicutes and shows vertical descent. However, ahrC also has a paralog, near argS and/or COG-arcA, in many Firmicutes (but not in *B. subtilis*). This paralog has an intermittent distribution, and it is not clear if this is due to HGT or gene loss. It is also not clear if the duplication event that created this paralog occurred within the evolutionary lineage of *B. subtilis* ahrC. If yes, then argR and ahrC are non-orthologs. Alternatively, the duplication may have occured on a separate lineage (e.g., duplication within lactic acid bacteria, and HGT to some Bacillus and to Staphylococcus), in which case the paralog is irrelevant to the status of *B. subtilis* ahrC, and argR/ahrC should be classified as xenologs because of the HGT in the *E. coli* lineage.

Conserved function, evolutionary xenologs

birA/birA – Both *E. coli* and *B. subtilis* birA are bifunctional, acting in the biotin synthesis pathway and as biotin operon repressors. As discussed in the text, birA appears to have undergone HGT between Bacilli and Archaea.

yjdG/yufM – yjdG is also known as dcuR, while yufM is also known as malR. dcuR regulates anaerobic fumurate respiration in response to various C4 acids including malate. (This is mediated by the histidine kinase dcuS.) yufM regulates malate transporters (5). These were classified as the same function because they respond to the same inducer, despite of some differences in biological role (e.g., *E. coli* does not take up malate under the conditions where dcuR has been studied (6). dcuR and yufM are related by a recent HGT event between Enterobacteria and Bacilli that involved their respective histidine kinases dcuS and yufL as well.

yjeB/yhdE – Both yjeB and yhdE are also known as nsrR. Both yjeB and yhdE regulate the response to nitrostative stress (7). yjeB is well-conserved within β,γ-Proteobacteria, and has been transferred to Bacilli to give yhdE (other Firmicutes lack yhdE).

Conserved function, evolutionary orthologs

dnaA/dnaA – Both regulate the initiation of DNA replication as well as their own transcription. There are some differences in the genes they regulate, however: *E. coli* dnaA regulates genes for DNA synthesis, while *B. subtilis* dnaA regulates sporulation. dnaA shows vertical descent within Firmicutes and within the β,γ-Proteobacteria, except perhaps for *Buchnera aphidicola* strains Sg and Bp. *Buchnera* are intracellular symbionts with accelerated evolution, so this probably reflects long-branch attraction rather than HGT.

fur/fur (*B. subtilis* fur is also known as yqkL) – Both respond to iron limitation and both regulate fepC/yusV, fepD/yfiZ, and entA/dhbA. fur shows vertical descent within β,γ-Proteobacteria and within Firmicutes.

lexA/lexA – Both regulate the SOS response. lexA shows vertical descent within γ-Proteobacteria and Firmicutes.

rpoN/sigL – These are also known as σ^{54}. Although there are several pathways that are known to be regulated by rpoN in *B. subtilis* but are not known in *E. coli*, these are primarily nitrogen-related (e.g., utilization of arginine and ornithine). rpoN shows vertical descent within γ-Proteobacteria. sigL shows mostly vertical descent in the Firmicutes, but it is absent from *Staphylococcus* species, and *Oceanobacillus iheyensis* seems to have acquired its rpoN from Clostridia. As the HGT event doesn't affect the *B. subtilis* lineage this was classified as an ortholog.

Different function, evolutionary non-orthologs

atoC/rocR – atoC controls acetoacetate metabolism, while rocR controls arginine utilization. atoC has undergone recent HGT and also has a closer paralog zraR (also known as hydG). rocR was acquired by HGT (it is absent from most Firmicutes outside of the Bacilli). atoC and rocR have

different N-terminal domains, so the method of (8) for identifying bidirectional best hits excludes this pair.

betI/pksA – betI responds to osmotic stress via choline, while pksA regulates a polyketide synthase operon. Both betI and pksA have undergone HGT (Figure 2).

cpxR/yycF – cpxR senses envelope stress, e.g. protein misfolding, while yycF is an essential regulator of membrane fatty acid synthesis. yycF is also phosphorylated in response to phosphate limitation (9). yycF has a closer paralog phoP. The older history of both genes is unclear.

cytR/ccpA – cytR regulates nucleoside utilization in response to cytidine levels, while ccpA mediates carbon catabolite repression, probably in response to NADP. cytR has been acquired recently, perhaps from *Geobacillus* or *Caulobacter*, and seems to be more closely related to *E. coli* purR and rbsR than to ccpA. ccpA has a closer paralog degA. As mentioned in the text, cytR/ccpA regulate BBH genes deoC/dra, but this seems to reflect convergent evolution rather than conservation of ancestral regulation. This is both because of the functional differences between cytR and ccpA and because of differences in the evolutionary histories. For example, cytR was acquired by HGT relatively recently: it is present in *Vibrio* species but not in more distant relatives of *E. coli*. In contrast, close relatives of *E. coli* deoC are found in most γ-Proteobacteria. It seems unlikely that the regulation of deoC by cytR predates them being in the same organism (e.g., the divergence of *E. coli* and *Vibrio*).

fecI/sigZ – fecI is a sigma factor that responds to fecR and activates genes for the transport of ferrous citrate. Little is known about sigZ, but overexpression of sigZ has no effect on the expression of genes for iron metabolism (10). fecI shows HGT between Enterobacteria, *Azotobacter*, and *Pseudomonas*, along with its sensor fecR and, in some cases, an adjacent regulated gene fecA. sigZ was recently acquired by HGT (its closest relatives are from outside the Bacilli).

glpR/glcR – glpR binds glycerol-3-phosphate and regulates glycerol utilization, while glcR regulates ptsG (glucose-specific phosphotransferase system) and helps determine carbon source choice (11). glpR has a closer paralog yihW (and possibly also deoR), while glcR was acquired by HGT: it has a close relatives in other Bacilli (and paralogs within Lactobacillales) but not in other Firmicutes.

lrp/azlB – lrp is a global regulator that responds to leucine, while azlB regulates the transport of branched-chain amino acids and appears to have a more specific function (see text). azlB has a complex history of HGT, with its closest relatives being in Clostridia, δ-Proteobacteria, and β-Proteobacteria. Also, lrp has closer paralogs than azlB in some γ-Proteobacteria, although not in *E. coli* – lrp is closer to *Pseudomonas putida* bkdR and to *Salmonella enterica Cholerasuis* tinR than to azlB, and both of these organisms also contain orthologs of lrp.

nagC/xylR – nagC responds to N-acetylglucosamine, while xylR responds to xylose. *E. coli* has a more closely related paralog, mlc, of unclear function, and their common ancestor was acquired

by HGT. xylR's history within the Firmicutes also involves a gene duplication (Figure 2).

phoB/resD – phoB responds to phosphate limitation via the histidine kinase phoR. resD regulates anerobic vs. anaerobic respiration. resD has several closer paralogs, and the resD/yclJ duplication seems to have occurred within Firmicutes (Figure 2). The older history of both genes is unclear.

rpoE/sigW – rpoE and sigW are sigma factors that are controlled by anti-sigma-factors (rseA and rsiW=ybbM, respectively). rseA and rsiW are in turn controlled by two-step proteolysis: rseA is cleaved by degS and and by yaeL=rseP, while rsiW is cleaved by yluC and by ypdC=prsW (12; 13). rseP and yluC are homologous, but the other components of the sensing system do not appear to be homologous by BLASTp or by InterPro domains. Furthermore, most of these components do not have any homologs (by BLASTp) in the other organism (e.g. rseA, rsiW, prsW). rseA activity may also be regulated by rseB and rseC, which also lack homologs in *B. subtilis*. Overall, the sensing pathways that control these sigma factors are significantly different. They also seem to sense different signals – degS (and perhaps also rseB) senses unfolded periplasmic proteins, while prsW is proposed to sense antimicrobial peptides. The pathways regulated by these sigma factors are also described as differing: rpoE activates a response to extracytoplasmic stress, while sigW activates the detoxification and also the synthesis of antimicrobial compounds. Because of the large number of genes regulated by rpoE, and the limited data on the sigW regulon, we are not sure if these functions overlap. Nonetheless, because of the many differences, we classified rpoE and sigW as having different functions. The evolution of rpoE shows vertical descent within the β,γ-Proteobacteria. Interestingly, the ortholog in *Pseudomonas aeruginosa*, known as algU or algT, regulates alginate production, which is important for pathogenesis in cystic fibrosis patients, in addition to regulating stress responses (14; 15). sigW has a complex history of HGT, e.g. it is present in Bacillacaea but not in most Firmicutes. sigW also has paralogs in *B. cereus* and *B. anthracis* that seem to have been acquired in a separate HGT event, but this many not affect the *B. subtilis* lineage.

hupA/hbs – hupA is also known as HU-α and HU-2. hbs is also known as HBsu. hupA, together with hupB, forms HU, a major "histone-like" nucleoid protein that may be involved in DNA compaction. HU also binds double-stranded RNA structures (16). Unlike hupA, hbs is essential for growth, and it is reported to be a component of the signal recognition particle (17). This interaction is mediated by a portion of the SRP RNA that is not present in *E. coli*, and seems to indicate a difference in function. Furthermore, we did not find evidence that hbs forms a heterodimer, and hupB does not have a BBH in *B. subtilis*. In fact, hupA and hupB are more closely related to each other than hupA is to hbs. Thus, hupA arose by gene duplication after the divergence from hbs. hbs also has a closer paralog yonN. The older history of both genes is unclear.

farR/yvoA – farR is also known as mngR. farR regulates the TCA cycle in response to fatty acid deficiency (it binds acyl-CoA), while yvoA regulates the non-essential methionine aminopeptidase yflG (18). farR shows multiple recent HGT events (e.g., it is absent from other Enterobacteria), and also has a closer paralog yidP.

yhcK/ycbG – yhcK is also known as nanR. yhcK regulates the response to sialic acid, while ycbG regulates the response to to galactarate and glucarate (19). Both yhcK and ycbG show recent HGT.

narP/degU – narP regulates nitrate/nitrite balance and anaerobic respiration, while degU regulates enzymes for degrading extracellular material. narP has a recent paralog narL, and has undergone HGT as well, while degU has a closer paralog yhcZ.

Different function, evolutionary xenologs

None

Different function, evolutionary orthologs

None

Unknown function in B. subtilis, evolutionary non-orthologs

cspA/cspC – *B. subtilis* cspC has recent paralogs cspB and cspD. *B. subtlis* cspB has a similar function as *E. coli* cspA in binding to single-stranded "Y-box" DNA, but we did not find any information as to whether cspC also has this function.

cynR/ywbI – cynR responds to cyanate. As discussed in Table 1, ntcB from *Synechoccus elongatus*, which is related to both genes, responds to nitrite (20), which suggests that cynR's function might not be conserved in ywbI. Both cynR and ywbI have a history of HGT.

egbR/msmR – ebgR responds to lactose and respresses a cryptic β-galactosidase. msmR has conserved proximity to genes for α-galactoside utilization, which suggests a somewhat different function. Given that msmR has a gene name, we searched for evidence to confirm this hypothesis. However, the gene name appears to derive from msmR of *Streptococcus mutans*, which is distantly related to both the *B. subtilis* and *E. coli* genes. Hence, msmR seems to be a misnomer for the *B. subtilis* gene. ebgR and msmR are closely related, but several recent HGT events seem to lie between them. For example, ebgR is absent from most Enterobacteria, and the gene tree suggests that it has been transferred from Vibrionaceae to *E. coli*. msmR is present in other members of *Bacillus* and also in *Clostridium* and *Geobacillus kaustophilus* (both Firmicutes) and also in *Sinorhizobium meiloti*, a β-Proteobacterium. Because multiple HGT events seem to separate ebgR and msmR (at least Firmicutes to Vibrionaceae to Escherichia), egbR/msmR were classified as non-orthologous rather than xenologous.

emrR/ykoM – We were not able to find any information on ykoM's function. emrR has a closer paralog papX in some strains of *E. coli.* Both emrR and ykoM have undergone multiple recent HGT events.

marR/yhbI – We were not able to find any information on ykoM's function. yhbI shows several recent HGT events.

narL/yhcZ – narL responds to nitrite/nitrate levels. yhcZ is closely related to vraR from Staphylococcus aureus (21), which is involved in regulating peptidoglycan synthesis rather than responding to nitrate/nitrite levels. narL has a closer paralog narP, while yhcZ has a closer paralog degU. yhcZ is absent from Firmicutes outside the genus Bacillus and was hence acqurid by HGT.

oxyR/ycgK – We were not able to find any information on ycgK's function. ycgK shows several recent HGT events.

yaeG/ysfB – yaeG is also known as cdaR and sdaR, and it regulates the metabolism of sugar diacids such as D-glycerate. We were not able to find any information on ysfB's function. yaeG shows both duplication and HGT within γ-Proteobacteria. It has a paralog in some Salmonella genomes, and the more distantly related Yersinia genus has the paralog instead of yaeG. It has a close homolog in Xanthomonas but not in most of the distant γ-Proteobacteria, which suggets HGT. ysfB shows recent HGT – it has close homologs in distant Firmicutes such as Moorella, and also in some γ-Proteobacteria (but not in *E. coli*).

ybbI/yraB – ybbI is also known as cueR, and it regulates copper homeostasis. yraB's function is unknown, although there is a report that, unlike some other members of this gene family in *B. subtilis*, yraB is *not* required for copper homeostasis (22). ybbI has characterized homologs with similar functions in *Rhizobium* and *Sinorhizobium* that are encoded by plasmids, which suggests a history of HGT, but this is not certain. yraB is absent from the other sequenced Bacillales and hence seems to have been acquired by HGT recently, perhaps from Enterococcus.

pepA/yuiA – pepA is a peptidase, is involved in site-specific recombination, and also regulates carAB and carP. We did not find any information on the function of yuiA. pepA is well-conserved within β,γ-Proteobacteria, while yuiA seems to have a complex history of HGT.

rhaR/yfiF – rhaR regulates rhamnose catabolism. We did not find any information on the function of yfiF. rhaR is a recent paralog of rhaS, and both rhaR and yfiF have a complex history.

rhaS/ybfI – rhaS regulates rhamnose catabolism. We did not find any information on the function of ybfI. rhaS is a recent paralog of rhaR, and both rhaS and ybfI have a complex history.

Unknown function in B. subtilis, evolutionary xenologs

None

Unknown function in B. subtilis, evolutionary orthologs

None

References

[1] Karp PD, Riley M, Saier M, Paulsen IT, Collado-Vides J, et al. (2002) The EcoCyc database. Nucleic Acids Res 30:56–8.

[2] Makita Y, Nakao M, Ogasawara N, Nakai K (2004) DBTBS: database of transcriptional regulation in Bacillus subtilis and its contribution to comparative genomics. Nucleic Acids Res 32:D75–7.

[3] Moszer I, Jones LM, Moreira S, Fabry C, Danchin A (2002) SubtiList: the reference database for the Bacillus subtilis genome. Nucleic Acids Res 30:62–5.

[4] Makarova KS, Mironov AA, Gelfand MS (2001) Conservation of the binding site for the arginine repressor in all bacterial lineages. Genome Biol 2:RESEARCH0013.

[5] Doan T, Servant P, Tojo S, Yamaguchi H, Lerondel G, et al. (2003) The Bacillus subtilis ywkA gene encodes a malic enzyme and its transcription is activated by the YufL/YufM two-component system in response to malate. Microbiology 149:2331–43.

[6] Zientz E, Bongaerts J, Unden G (1998) Fumarate regulation of gene expression in Escherichia coli by the DcuSR (dcuSR genes) two-component regulatory system. J Bacteriol 180:5421–5.

[7] Nakano MM, Geng H, Nakano S, Kobayashi K (2006) The nitric oxide-responsive regulator NsrR controls ResDE-dependent gene expression. J Bacteriol 188:5878–87.

[8] Lozada-Chavez I, Janga SC, Collado-Vides J (2006) Bacterial regulatory networks are extremely flexible in evolution. Nucleic Acids Res 34:3434–45.

[9] Howell A, Dubrac S, Noone D, Varughese KI, Devine K (2006) Interactions between the YycFG and PhoPR two-component systems in Bacillus subtilis: the PhoR kinase phosphorylates the non-cognate yycf response regulator upon phosphate limitation. Mol Microbiol 59:1199–215.

[10] Asai K, Yamaguchi H, Kang CM, Yoshida K, Fujita Y, et al. (2003) DNA microarray analysis of Bacillus subtilis sigma factors of extracytoplasmic function family. FEMS Microbiol Lett 220:155–60.

[11] Stulke J, Martin-Verstraete I, Glaser P, Rapoport G (2001) Characterization of glucose-repression-resistant mutants of Bacillus subtilis: identification of the glcR gene. Arch Microbiol 175:441–9.

[12] Ellermeier CD, Losick R (2006) Evidence for a novel protease governing regulated intramembrane proteolysis and resistance to antimicrobial peptides in Bacillus subtilis. Genes Dev 20:1911–22.

[13] Schobel S, Zellmeier S, Schumann W, Wiegert T (2004) The Bacillus subtilis sigmaW anti-sigma factor RsiW is degraded by intramembrane proteolysis through YluC. Mol Microbiol 52:1091–105.

[14] DeVries CA, Ohman DE (1994) Mucoid-to-nonmucoid conversion in alginate-producing Pseudomonas aeruginosa often results from spontaneous mutations in algT, encoding a putative alternate sigma factor, and shows evidence for autoregulation. J Bacteriol 176:6677–87.

[15] Martin DW, Schurr MJ, Yu H, Deretic V (1994) Analysis of promoters controlled by the putative sigma factor AlgU regulating conversion to mucoidy in Pseudomonas aeruginosa: relationship to sigma E and stress response. J Bacteriol 176:6688–96.

[16] Balandina A, Kamashev D, Rouviere-Yaniv J (2002) The bacterial histone-like protein HU specifically recognizes similar structures in all nucleic acids. DNA, RNA, and their hybrids. J Biol Chem 277:27622–8.

[17] Nakamura K, Yahagi S, Yamazaki T, Yamane K (1999) Bacillus subtilis histone-like protein, HBsu, is an integral component of a SRP-like particle that can bind the Alu domain of small cytoplasmic RNA. J Biol Chem 274:13569–76.

[18] You C, Lu H, Sekowska A, Fang G, Wang Y, et al. (2005) The two authentic methionine aminopeptidase genes are differentially expressed in Bacillus subtilis. BMC Microbiol 5:57.

[19] Hosoya S, Yamane K, Takeuchi M, Sato T (2002) Identification and characterization of the Bacillus subtilis D-glucarate/galactarate utilization operon ycbCDEFGHJ. FEMS Microbiol Lett 210:193–9.

[20] Aichi M, Maeda S, Ichikawa K, Omata T (2004) Nitrite-responsive activation of the nitrate assimilation operon in cyanobacteria plays an essential role in up-regulation of nitrate assimilation activities under nitrate-limited growth conditions. J Bacteriol 186:3224–9.

[21] Kuroda M, Kuroda H, Oshima T, Takeuchi F, Mori H, et al. (2003) Two-component system VraSR positively modulates the regulation of cell-wall biosynthesis pathway in Staphylococcus aureus. Mol Microbiol 49:807–21.

[22] Gaballa A, Cao M, Helmann JD (2003) Two MerR homologues that affect copper induction of the Bacillus subtilis copZA operon. Microbiology 149:3413–21.
