# Supplementary material for: Orthologous Transcription Factors in Bacteria Have Different Functions and Regulate Different Genes
Source: PLoS Comput Biol. 2007 Sep 7;3(9):e175. doi: 10.1371/journal.pcbi.0030175 (PMC1971122; doi:10.1371/journal.pcbi.0030175)
Supplement: Supplementary file 2 [file pcbi.0030175.sd002.pdf]

Supplementary Text S2: Differences in function for other BBHs

Madan Babu & Teichmann (1) propose that the sensing capabilities of crp and fnr (for cAMP and oxygen, respectively) have been conserved since the divergence of the prokaryotes. Lozada-Chavez and colleagues (2) also suggest that crp and fnr have "highly conserved regulons." However, crp and fnr belong to a large family of transcription factors that respond to a variety of signals. In the case of crp, cAMP sensing has only been documented for a subfamily that is restricted to *E. coli* and other γ-Proteobacteria (3), and the BBH of crp in the cyanobacterium *Synechococcus elongatus* is ntcA (NTCA_SYNP7), which regulates nitrogen assimilation (not carbon source utilization) and binds 2-oxoglutarate (not cAMP) (4). For fnr, we note that its BBH in the α-Proteobacterium *Caulobacter cresentus* CB15 is CC0752 (fixK). Whereas fnr responds to O_2 levels directly and upregulates genes that are required for anaerobic growth, fixK responds to O_2 levels indirectly, via the two-component system fixLJ, and upregulates high-affinity oxidases (5). Finally, the suggestion that the crp/fnr family is present in Archaea has been disputed, because key functional residues are not conserved in the putative family members (3). Consistent with this, the 26 sequenced Archaea in MicrobesOnline (6) do not contain any genes assigned to COG664, which contains crp, fnr, and fixK.

Madan Babu & Teichmann (1) suggest that lrp is an ancient regulatory hub. In *E. coli*, lrp is a global regulator that responds to leucine levels. The BBH in *B. subtilis* is azlB, which regulates the transport of branched-chain amino acids (7). azlB and the adjacent gene azlC, which encodes a transporter that is regulated by azlB, have undergone repeated HGT together (their closest relatives are from several different divisions, with the genes adjacent). Thus, azlB probably has a specific function relating to azlC. Also, azlB is less closely related to lrp than it is to some γ-Proteobacterial paralogs of lrp, such as tinR from *Salmonella enterica Choleraesuis* and bkdR from *Pseudomonas putida* (data not shown). Furthermore, lrp's role as a global regulator is not conserved in *Haemophilus influenzae* (8), which is much more closely related to *E. coli* than *B. subtilis*, and other characterized members of the lrp family are not global regulators (9). Overall, it seems unlikely that lrp was a global regulator in ancestral bacteria or archaea.

Another example highlighted by (1) is a feed-forward circuit of the global regulator fnr, the response regulator narL, and NADH dehydrogenase component nuoN, in which fnr regulates narL and both fnr and narL regulate nuoN. They propose that this entire regulatory system, which has been characterized in *E. coli*, is present in distantly related organisms such as the α-Proteobacterium *Rhodospeudomonas palustris*, even though it is absent from most organisms of intermediate relatedness. *E. coli* narL responds to nitrate and nitrite via the response regulators narQ and narX, and they predict that narL is orthologous to *R. palustris* genbank index 22963462 (or NP_946837). This gene appears to be in an operon (10) with a bacteriophytochrome (a light-sensing histidine kinase, COG4251). As histidine kinases are very often adjacent to or cotranscribed with the response regulators that they control (e.g., data of (11)), and because a histidine kinase and response regulator are unlikely to be adjacent by chance, *R. palustris* "narL" probably responds to light rather

than nitrate levels. In support of this view, most of the close relatives of NP_946837 are adjacent to histidine kinases (not necessarily phytochrome-containing). Also, NP_946837 is phylogenetically more closely related to *B. subtilis* ydfI than to *E. coli* narL or to narL's paralog narP (data not shown). ydfI is regulated by an adjacent histidine kinase and in turn regulates transcription of ydfJ, but its biological role is not known (12). In any case, it seems unlikely that *R. palustris* NP_946837 responds to nitrate levels or regulates nuoN.

References

[1] Babu MM, Teichmann SA, Aravind L (2006) Evolutionary dynamics of prokaryotic transcriptional regulatory networks. J Mol Biol 358:614–33.

[2] Lozada-Chavez I, Janga SC, Collado-Vides J (2006) Bacterial regulatory networks are extremely flexible in evolution. Nucleic Acids Res 34:3434–45.

[3] Körner H, Sofia HJ, Zumft WG (2003) Phylogeny of the bacterial superfamily of Crp-Fnr transcription regulators: exploiting the metabolic spectrum by controlling alternative gene programs. FEMS Microbiology Reviews 27:559–592.

[4] Tanigawa R, Shirokane M, Omata S, Omata T, Tanaka K, et al. (2002) Transcriptional activation of NtcA-dependent promoters of Synechococcus sp. PCC 7942 by 2-oxoglutarate in vitro. Proc Natl Acad Sci U S A 99:4251–5.

[5] Crosson S, McGrath PT, Stephens C, McAdams HH, Shapiro L (2005) Conserved modular design of an oxygen sensory/signaling network with species-specific output. Proc Natl Acad Sci U S A 102:8018–23.

[6] Alm EJ, Huang KH, Price MN, Koche RP, Keller K, et al. (2005) The MicrobesOnline web site for comparative genomics. Genome Res 15:1015–22.

[7] Belitsky BR, Gustafsson MC, Sonenshein AL, Wachenfeldt CV (1997) An lrp-like gene of Bacillus subtilis involved in branched-chain amino acid transport. J Bacteriol 179:5448–57.

[8] Friedberg D, Midkiff M, Calvo JM (2001) Global versus local regulatory roles for Lrp-related proteins: Haemophilus influenzae as a case study. J Bacteriol 183:4004–11.

[9] Brinkman AB, Ettema TJG, de Vos WM, van der Oost J (2003) The Lrp family of transcriptional regulators. Mol Microbiol 48.

[10] Price MN, Huang KH, Alm EJ, Arkin AP (2005) A novel method for accurate operon predictions in all sequenced prokaryotes. Nucleic Acids Res 33:880–92.

[11] Yamamoto K, Hirao K, Oshima T, Aiba H, Utsumi R, et al. (2005) Functional characterization in vitro of all two-component signal transduction systems from Escherichia coli. J Biol Chem 280:1448–1456.

[12] Serizawa M, Sekiguchi J (2005) The Bacillus subtilis YdfHI two-component system regulates the transcription of ydfJ, a member of the RND superfamily. Microbiology 151:1769–78.
