# Supplementary material for: Orthologous Transcription Factors in Bacteria Have Different Functions and Regulate Different Genes
Source: PLoS Comput Biol. 2007 Sep 7;3(9):e175. doi: 10.1371/journal.pcbi.0030175 (PMC1971122; doi:10.1371/journal.pcbi.0030175)
Supplement: Supplementary file 3 [file pcbi.0030175.sd003.pdf]

Supplementary Text S3: Regulatory differences between E. coli and S. oneidensis MR-1

We examined genes that have gene-regulation correlations above 0.5 in *E. coli* K12 but whose BBHs have gene-regulation correlations below 0 in *S. oneidensis* MR-1. For each example, we report any evidence that the regulation has changed (or not). If any of the regulators or the regulated genes are not evolutionary orthologs, this is also mentioned.

We also tried to identify binding sites for arcA, argR, fur, rpoE, and rpoH. We began with aligned sites from DPInteract (1), and used MEME (2) to extract a motif (a range of informative positions and a subset of well-matching sequences). We used patser (3) to use the resulting weight matrix to search both strand of upstream regions. (For the σ-factors rpoE and rpoH we used only the coding strand.) We searched from 200 bases upstream to 100 bases downstream of the first start codon in each operon. For rpoH, which has two weight matrices for different spacings, we report the higher of the two scores. We did not use the rpoE weight matrix because it is not specific. We did not search for fliA sites because DPInteract does not include an alignment for this TF and we did not find a strong motif for its regulon in *E. coli*.

To determine if potential binding sites in *Shewanella oneidensis* MR-1 were conserved in other species of Shewanella, we examined orthologous upstream regions in *S.* PV-4, *S. amazonensis* SB2B, *S. baltica* OS155, *S. denitrificans* OS217, *S. frigidimarina* NCIMB 400, *S. putrefaciens* CN-32, *S.* W3-18-1, *S.* MR-4, *S.* MR-7, and *S.* ANA-3. Sites were considered conserved if a putative site with similar sequence and a similar or greater score was found in at least one of these other genomes.

arcA

A number of the genes with changed expression patterns are regulated by arcA in *E. coli*. arcA seems to have a somewhat different biological role in *S. oneidensis* than in *E. coli*. In particular, the signal transduction mechanism leading to arcA contains different components. In *E. coli*, arcA accepts phosphates from the histidine kinase arcB, but the *S. oneidensis* genome does not encode arcB; instead, in *S. oneidensis*, arcA accepts phosphates from the phosphotransfer protein hptA (4). hptA is homologous to the N-terminal Hpt domain of arcB, and hptA probably receives its phosphate from an as-yet-unidentified histidine kinase. Other reported differences are that arcA induces the high-affinity cytochrome oxidase cydAB under anaerobic conditions in *E. coli* but represses cydAB in *S. oneidensis* MR-1, and that arcA regulates the sdh operon in *E. coli* but not in *S. oneidensis* (4). These differences exist even though the arcA protein itself is very similar in the two organisms: the protein sequences are over 80% identical, and *S. oneidensis* MR-1 arcA complements an arcA knockout in *E. coli* (4).

arcA, crp, fnr, & fur → sdhAB: known change. The response regulator arcA regulates the sdhCDAB-sucABCD operon in *E. coli*, together with several other regulators. Of the genes in this operon, the automated microarray analysis found changed expression patterns for sdhA and sdhB. ArcA does not regulate this operon in *S. oneidensis* (4). More precisely, arcA is required for anaerobic repression of the sdh operon in *E. coli* (5), but knocking out arcA has no effect on the anaerobic repression of sdhC in *S. oneidensis*. Sequence analysis of potential arcA binding sites was inconclusive (data not shown).

arcA, crp, fnr, & fur → cyoE: false ortholog, and change in operon structure. The *S. oneidensis* BBH of cyoE, named ctaB, is distantly related to *E. coli* cyoE and has a complex evolutionary history (data not shown). Because cyoE and ctaB are separated by HGT events between distant organisms, it seems unlikely that the regulation has been conserved. Also, *E. coli* cyoE is within the operon cyoABCDE, while *S. oneidensis* ctaB is within a larger functional cluster of genes cyoAB-coxG-cyoC-(SO4610)-COG3346-SO4612-ctaA-ctaB-COG1999. (cyoD appears to be absent from *S. oneidensis*.) Because SO4610 is on the opposite strand, and because SO4610 seems to be a genuine gene (it is conserved in other Shewanellas and in some Vibrios), it seems that ctaB is not cotranscribed with cyoAB-coxG-cyoC. Sequence analysis of potential arcA binding sites was inconclusive (data not shown).

arcA & fnr → rpsJ-rplCDWB-rpsS-rplV-rpsC-rplP-rpmC-rpsQ: unclear. This large operon, which mostly encodes ribosomal proteins, is regulated by arcA and fnr in *E. coli*, but the regulation by fnr is inferred from expression analysis (6) and might be indirect. These genes are in the same order in *S. oneidensis*, and all of the genes except for rpmC were identified as having changed expression patterns in MR-1. fusA-tufB are upstream of rpsJ in *S. oneidensis* and many other γ-Proteobacteria, so the operon might be longer and expressed from a different promoter in *S. oneidensis*. The known changes in arcA function in *S. oneidensis* discussed above also suggest that this regulation might have changed. Sequence analysis of potential arcA binding sites was inconclusive (data not shown).

arcA & fnr → tpx: loss of predicted site. *S. oneidensis* tpx is distantly related to *E. coli* tpx, and several HGT events separate the two genes. A strong arcA binding site was predicted upstream of *E. coli* arcA but not upstream of *S. oneidensis* MR-1 arcA (data not shown).

argR

argR → argC & argH: change in operon structure and predicted loss of binding site. In *E. coli*, argR regulates the operon argCBH and also the divergently transcribed gene argE from two shared binding sites. In *S. oneidensis* MR-1, the gene cluster has expanded to argCBFGH, all on the same strand, and argE has been lost. We searched for but did not find a clear argR site upstream of argC (the best hit is 6.1 bits, and it is not conserved in other Shewanellas). In contrast, there is a strong prediction for a conserved site upstream of argB (agTGcATaaagATtCAct, 11.4

bits). The putative argB site is one of the strongest hits to the argR weight matrix in the MR-1 genome, and given this and the functional relationship, it seems very unlikely to be a false positive. This suggests that argB has its own promoter and that argC is expressed separately (and apparently is not regulated by argR). As expected given the gene-regulon correlation, the expression of argC and argH is not correlated with that of argBFG ($r \approx 0$), but argC and argH are strongly correlated with each other ($r = 0.88$). To explain the expression pattern of argH, we speculate that it also has its own promoter.

fliA

fliA → fliN & fliQ: known change, false orthology, and change in operon structure In *E. coli*, fliN and fliQ are expressed from the operon fliLMNOPQR, which is transcribed from a fliA-dependent promoter. (FliA is also known as σ^{28}.) The *E. coli* operon also has a σ^{70}-dependent promoter that is regulated by flhDC, but as *S. oneidensis* does not appear to contain either flhD or fhlC, so we will not consider that further. The *S. oneidensis* MR-1 flagellar operons have not, as far as we know, been characterized yet, but the system has been studied in *Vibrio parahaemolyticus*. The genes for the polar flagellum of *V. parahaemolyticus* are closely related to the *S. oneidensis* flagellar genes, while the *E. coli* flagellar genes are more distant and have undergone multiple HGT events (data not shown). Thus, fliA, fliN and fliQ are not evolutionary orthologs between *E. coli* and *S. oneidensis*, and any difference in regulation between *E. coli* and *V. parahaemolyticus* is likely to apply to *S. oneidensis* as well. In *V. parahaemolyticus*, fliN and fliQ are part of a larger operon fliEFGHIJKLMNOPQR-flhB which has a σ^{54}-dependent promoter and is not regulated by fliA (7). *S. oneidensis* has the same gene order as *V. parahaemolyticus*.

fnr

Fnr seems to have a somewhat different role in *S. oneidensis*, where it is known as etrA. Whereas *E. coli* fnr was originally identified because it is required for growth with nitrate or fumarate as electron acceptors (8), an etrA- strain of *S. oneidensis* grows on a wide variety of electron acceptors, including fumarate and nitrate (9).

fnr → rpsP, rimM, & trmD: unclear, potential error in RegulonDB. (rimM is also known as yfjA.) In *E. coli*, these genes are contranscribed together with rplS in the operon rpsP-rimM-trmD-rplS. The regulation of rpsP by fnr is inferred from gene expression analysis and might be indirect (6). We did not find candidate fnr binding sites upstream of rpsP in either *E. coli* or *S. oneidensis* (data not shown). The gene order is conserved in *S. oneidensis*.

fnr → rplT: unclear, potential error in RegulonDB. The regulation is inferred from expression analysis and might be indirect (6). We did not find candidate fnr binding sites upstream of rplT in

either *E. coli* or *S. oneidensis* (data not shown).

rpoE

rpoE → fusA: unclear. In *E. coli*, fusA is transcribed together with the downstream gene tufA from a rpoE-dependent promoter, as well as from a promoter for the upstream genes rpsLG. (rpoE is also known as σ^{24}.) We do not have an effective model of rpoE binding sites. The gene order is conserved in *S. oneidensis*.

rpoE → lptB & rpoN: unclear. (lptB is also known as yhbG or b3201.) EcoCyc and RegulonDb report that the operon lptB-rpoN-ybhH-ptsN-yhbJ is expressed from an rpoE-dependent promoter. This promoter might actually lie upstream of lptA, also known as yhbH (10). We do not have an effective model of rpoE binding sites. The gene order is conserved in *S. oneidensis*.

rpoE → yfiO: unclear. (yfiO is also known as b2595.) yfiO is transcribed independently and has conserved gene order between *E. coli* and *S. oneidensis*. We do not have an effective model of rpoE binding sites.

rpoH

rpoH → clpP & clpX: predicted loss of binding site. In *E. coli*, the clpP-clpX operon is strongly activated by heat shock, due to a rpoH-dependent promoter. In *S. oneidensis* MR-1, however, clpP and clpX are slightly downregulated in response to heat shock (11). Also, we did not find any apparent rpoH binding site upstream of either clpP or clpX in *S. oneidensis* MR-1. Gao et al. note a potential site, but it seems very weak (it has an unusually narrow spacing between the -10 and -35 boxes and lacks the highly conserved C in the -10 box cCccatnt).

References

[1] Robison K, McGuire AM, Church GM (1998) A comprehensive library of DNA-binding site matrices for 55 proteins applied to the complete Escherichia coli K-12 genome. J Mol Biol 284:241–54.

[2] Bailey TL, Elkan C (1995) Unsupervised learning of multiple motifs in biopolymers using expectation maximization. Machine Learning 21:51–80.

[3] Hertz GZ, Stormo GD (1999) Identifying DNA and protein patterns with statistically significant alignments of multiple sequences. Bioinformatics 15:563–77.

[4] Gralnick JA, Brown CT, Newman DK (2005) Anaerobic regulation by an atypical Arc system in Shewanella oneidensis. Mol Microbiol 56:1347–57.

[5] Shen J, Gunsalus RP (1997) Role of multiple ArcA recognition sites in anaerobic regulation of succinate dehydrogenase (sdhCDAB) gene expression in Escherichia coli. Mol Microbiol 26:223–36.

[6] Salmon K, Hung SP, Mekjian K, Baldi P, Hatfield GW, et al. (2003) Global gene expression profiling in Escherichia coli K12. the effects of oxygen availability and FNR. J Biol Chem 278:29837–55.

[7] McCarter LL (2001) Polar flagellar motility of the Vibrionaceae. Microbiol Mol Biol Rev 65:445–62.

[8] Lambden PR, Guest JR (1976) Mutants of Escherichia coli K12 unable to use fumarate as an anaerobic electron acceptor. J Gen Microbiol 97:145–60.

[9] Maier TM, Myers CR (2001) Isolation and characterization of a Shewanella putrefaciens MR-1 electron transport regulator etrA mutant: Reassessment of the role of EtrA. J Bacteriol 183:4918–4926.

[10] Rhodius VA, Suh WC, Nonaka G, West J, Gross CA (2006) Conserved and variable functions of the sigmaE stress response in related genomes. PLoS Biol 4:e2.

[11] Gao H, Wang Y, Liu X, Yan T, Wu L, et al. (2004) Global transcriptome analysis of the heat shock response of Shewanella oneidensis. J Bacteriol 186:7796–803.
